# Supplementary figures and images for: A double‐blind, sham‐controlled, trial of home‐administered rhythmic 10‐Hz median nerve stimulation for the reduction of tics, and suppression of the urge‐to‐tic, in individuals with Tourette syndrome and chronic tic disorder
Source: J Neuropsychol. 2023 May 3;17(3):540–63. doi: 10.1111/jnp.12313 (PMC10947020; doi:10.1111/jnp.12313)

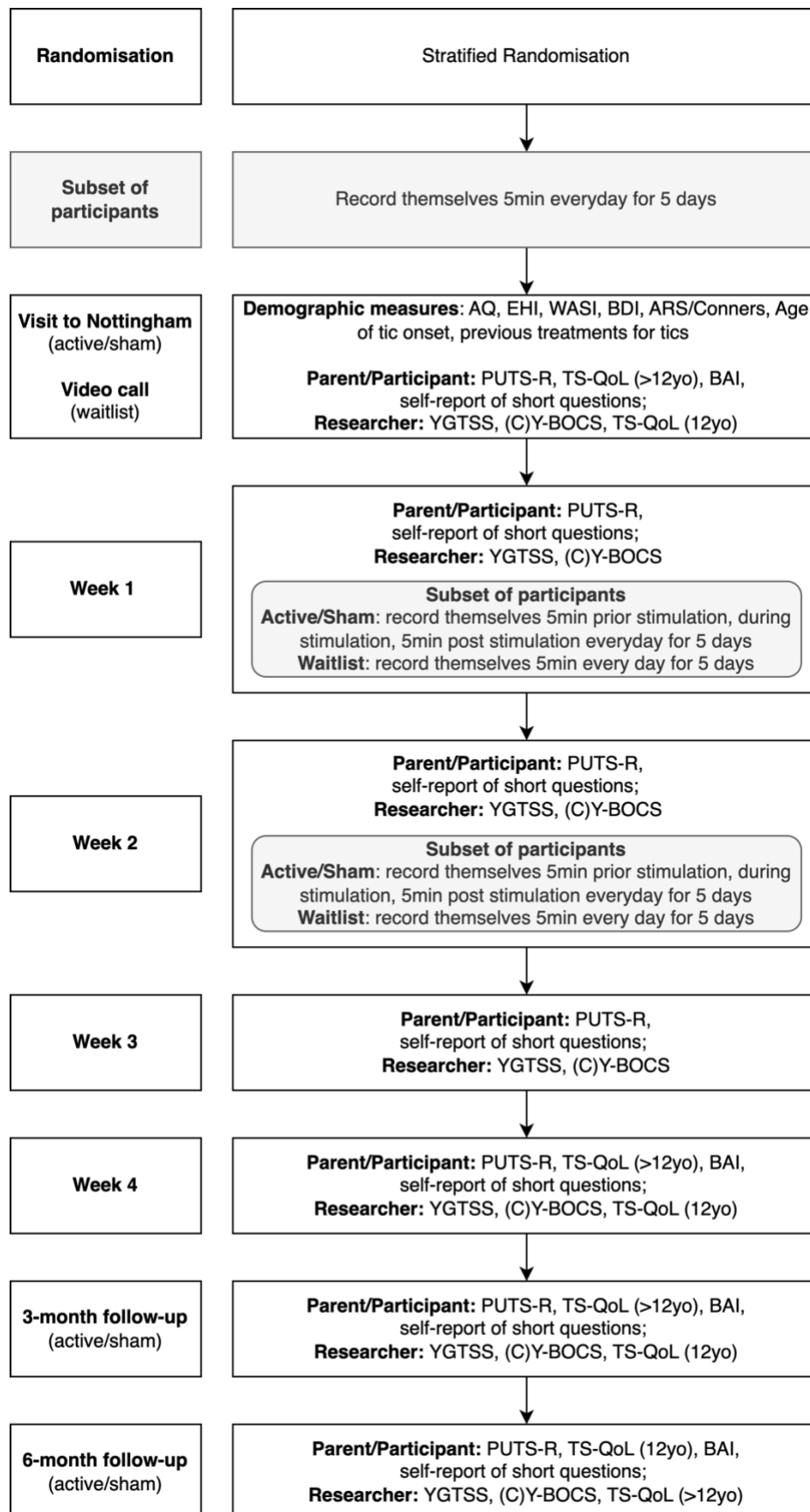

Figure 1: schematic flow diagram illustrating the timeline for collecting assessments

Supplement: Supplementary file 1 — Figure S1. [file JNP-17-540-s001.pdf]
